# Housing Supply and How It Is Related to Social Inequalities—Air Pollution, Green Spaces, Crime Levels, and Poor Areas—In Catalonia

**DOI:** 10.3390/ijerph20085578

**Published:** 2023-04-19

**Authors:** Xavier Perafita, Marc Saez

**Affiliations:** 1 Observatori—Organisme Autònom de Salut Pública de la Diputació de Girona (Dipsalut), 17003 Girona, Spain; 2 Research Group on Statistics, Econometrics and Health (GRECS), University of Girona, 17003 Girona, Spain; 3 CIBER of Epidemiology and Public Health (CIBERESP), 28029 Madrid, Spain

**Keywords:** inequality, housing poverty, NDVI, pollutants, poverty trap, life limitations

## Abstract

We carried out a search of over 12,000 houses offered on the rental market in Catalonia and assessed the possibility of families below the poverty threshold being able to rent these homes. In this regard, we wanted to evaluate whether the economic situation of families is able to influence their social environment, surroundings, and safety. We observed how their economic situation can allow families the possibility of developing a life without exposure to health risks, and how economic constraints result in disadvantages in several areas of life. The results show how families at risk of poverty live in less favourable conditions and experience a widening of different gaps, with current prices leading to a possible poverty trap for the most disadvantaged groups. The higher the percentage of the population below the threshold, the lower the possibility of not being able to rent a house compared to areas with a lower prevalence of population below the threshold. This association was observed both when considering the risk linearly and non-linearly. Linearly, the probability of not renting a house was reduced by 8.36% for each 1% increase in the prevalence of population at risk of extreme poverty. In the second, third and fourth percentage quartiles, the probability of not being able to rent a house decreased by 21.13%, 48.61%, and 57.79%, respectively. In addition, the effect was different inside and outside of metropolitan areas, with the former showing a decrease of 19.05% in the probability of renting a house, whereas outside metropolitan areas the probability increased by 5.70%.

## 1. Introduction

Social inequality can be viewed from different perspectives. These can be political, economic, social, or health-related, among others, and they can be interrelated [1,2,3,4,5]. Consequently, inequality culminates in the generation of situations of marginalisation and social exclusion. This problem is highlighted by the policies pursued by governments and institutions to try to minimalize these inequalities.

If we focus on income inequalities, there are several policies pursued in Europe to minimise the gap including minimum wages, taxes for redistributive purposes, and the establishment of welfare that guarantees minimum services for the whole population. As Piketty shows, the growth of economies alone does not reduce inequalities stemming from income and wealth [6]. These inequalities originate from the poor distribution of wealth among the population. However, the discourse around this line of thought does not revolve around an element that is common in the lives of everyone: housing.

Housing is one of the inequalities generated within the economic sphere and encompasses under-housing, housing deficiencies, unhealthy households, sub-renting, employment, unhealthy environments, and the cost of housing; all of which are elements that lead to social exclusion and other inequalities. Housing was declared a universal right in 1948 in the Universal Declaration of Human Rights (article 25), and later in the International Covenant on Economic, Social and Cultural Rights. In fact, it is one of the economic, social, and cultural rights with the greatest impact. Irrespective of the debate as to whether housing is a right or an obligation of governments [7], the fact is that there is a part of the population in situations of vulnerability with respect to homes [8].

Not all homes are optimal for developing a dignified and healthy lifestyle [9,10]. While most countries have their own regulations that determine what these minimums should be, there is still a significant gap between what is understood as a decent home [10] and each country’s domestic market prices.

This difference in prices and housing features causes a mismatch between the possibilities of accessing a decent home for different social classes. Although homes are one of the few financial assets most likely to be distributed among the middle classes, not all families can access one [11]. Those who can access a home must be alert to what percentage of their income is allocated to paying for it. If this percentage is very high, the pressure on the household may increase, having a negative effect on the social and economic life and health of its members. This is the reason why rent subsidies are provided in various countries [12,13]. The fact that house prices are not within the means of all families means that those with fewer financial resources cannot buy a home and must rent a property instead [14]. It is recommended that, whether for purchase or rental, the percentage of household income allocated to a home does not exceed 40% of the total income. If this percentage is not adhered to, one can more easily fall into the poverty trap. In 2015, 11.3% of the population living in the European Union lived in households that spent more than 40% of their family income on a home [15]. In 2018, 10.2% of households [16] at risk of poverty spent more than this threshold figure. Although this ceiling is generally 40%, EU countries adopt different thresholds ranging from 30% to 40% [16].

These features are also directly related to price. The type of housing and installations and the greater the number of square metres, toilets, and rooms it has, the higher its price will be. The area where the home is located must also be considered. If it is in an unsafe neighbourhood with a higher crime rate or there are fewer facilities nearby, the price drops significantly [17,18,19].

Another factor to consider is whether or not the area surrounding the home has green zones, including parks, forests, gardens, and trees, which are all linked to better health [20,21,22,23,24] and the acquisition of healthy habits [25]. Proximity to green spaces also affects the final price of a house, and so the further a house is from a green area, the lower its price. That said, the size of the green areas within the vicinity of the house does not seem to affect its final price [26]. Likewise, living in an area with high-density traffic or high levels of pollution will also have a direct impact on people’s quality of life [27]. This is also reflected in the price of a home whereby the greater the levels of pollution are (whether due to noise or air quality), the lower the price of the home will be [28,29,30].

Therefore, a low home price could be linked to a poorer quality home. This trend can become a cyclical problem, where lower income families are not able to access a large variety of homes. In addition, the properties to which they will have access will be those with a worse set of features, implying that they cannot access quality neighbourhoods in safer areas or those which provide a better quality of life.

Consequently, we can interpret the home as a key element in the development of people, right from childhood itself, and a key factor in the growth and reduction of inequalities in the medium and long terms.

In Spain, economic growth has been closely linked to the construction of private homes [31]. This growth has transformed households, considered a first-necessity good, into a speculative good. This speculation has resulted in a decrease in the number of households that can be accessed by low-income families.

On a demographic level, Catalonia has a unique structure in that more than 21% of its population lives in Barcelona; the second most populous city in Spain. Furthermore, more than 41% of its population lives either in Barcelona or one of its 36 adjacent towns, meaning that 41% of the population occupies just 1.97% of the region. By the same token, 66% reside in cities with more than 20,000 inhabitants which, in turn, is equivalent to just 6.61% of the total territory [32].

This rural/urban dichotomy has direct implications for inequality, and in those that affect living conditions above all. Urbanization has been present in many areas of the Catalan territory at the same time as inequalities have been accentuated and residential segregation manifested [33,34]. Recently, house prices have generally increased, especially in the metropolises [35,36,37]. In 2020, rental prices had increased so dramatically in Catalonia that a law was passed to regulate them. This law allows rents to be capped based on the Catalan Housing Agency’s Average Price Index, which sets an average reference price for specific areas where abusive price increases have been identified. In addition, with the appearance of homes destined for short-term rental platforms such as Airbnb, the number of homes available for rent has been reduced. This reduction is even greater in popular tourist areas and has resulted in a 7% increase in rental prices [38,39].

These circumstances end up affecting those with fewer economic resources as they must allocate a larger part of their income to cover housing, often at the expense of living in a healthy environment [40]. This paper aims to study the conditions under which families who are below the poverty threshold can rent a home. Our hypothesis is that the socially excluded cannot rent a property with conditions that ensure an optimal standard of living. We also assume that to have a home to live in, low-income families must interact with environments of high social inequality.

Our objective in this work is to study the ability of people at risk of social exclusion to access the rental housing market in Catalonia at the small scale (either municipality or district). Specifically, we evaluated the association between the possibility that a family at risk of exclusion does not rent a home and the type of socio-economic environment, the nearby urban greenery (green areas per inhabitant in square meters, the Normalized Difference Vegetation Index (NDVI) in a range of 500 m), the level of exposure to air pollution (PM_10_, NO_2_ and O_3_), and the level of known crimes in the area.

## 2. Materials and Methods

### 2.1. Area and Period of Study

We used a cross-sectional observational design, and the data were extracted from multiple databases in September 2021 to study accessibility. The area of study consisted of the geolocated information of each home combined with the information from the small areas (either municipality or district), to enable access to the most granular information possible. The study is based on data from households for rent in September 2021.

**Figure 1:**
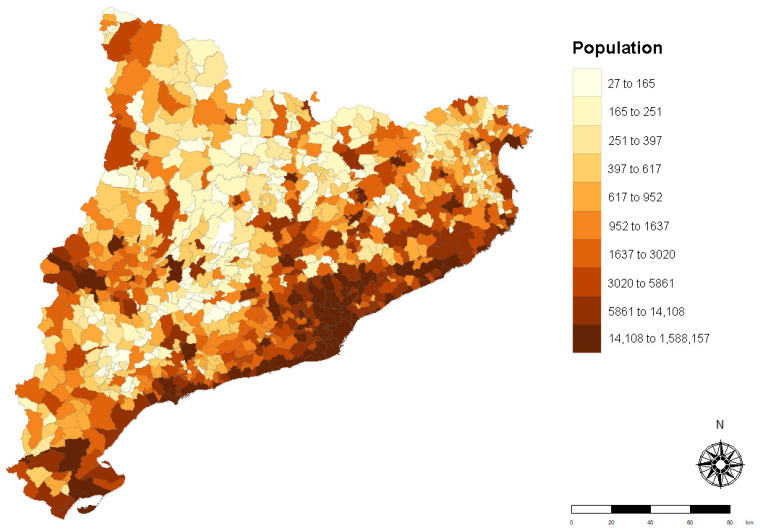
Map of the population of Catalonia by municipalities.

**Figure 2:**
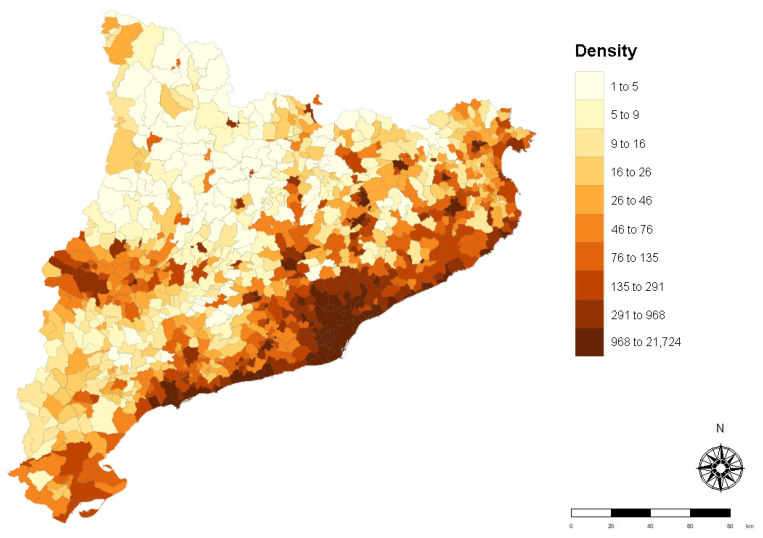
Map of the density of Catalonia by municipalities.

**Figure 3:**
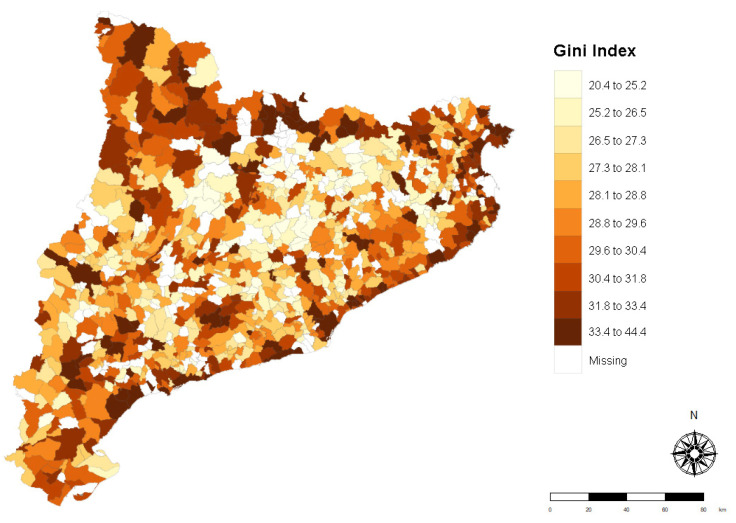
Map of the Gini index of Catalonia by municipalities.

**Figure 4:**
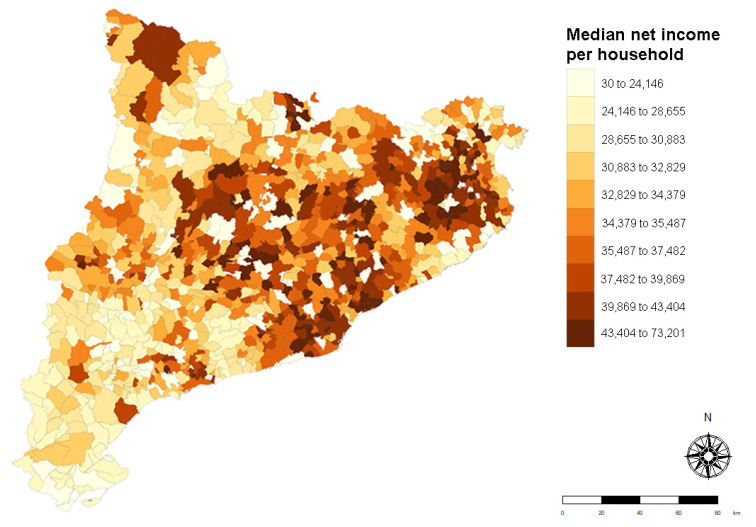
Map of the median net income per household of Catalonia by municipalities.

In Catalonia, as in the rest of Spain, only municipalities with 75,000 inhabitants or more are divided into districts. The districts, although they are an administrative division, group neighbourhoods with noticeable homogeneity in terms of socioeconomic variables (similar population density, similar disposable income, similar inequality index, etc.), see Figure 1, Figure 2, Figure 3 and Figure 4. In addition, the districts are the smallest administrative unit for which information is obtained for the variables of interest in this study (i.e., housing).

### 2.2. Methods Prior to Executing the Study, the Dataset, and Data Sources

#### 2.2.1. Data Sources

The data used were extracted from different sources, all of them official, and translated into a dataset of more than 50 variables (see Supplementary Table S1). The sources used to carry out the study were: Statistical Institute of Catalonia (IDESCAT), Cartographic and Geological Institute of Catalonia (ICGC), Government of Catalonia Open Data, the Mossos d’Esquadra police force, the National Statistics Institute (INE), Habitaclia estate agency and the Research Group on Statistics, Econometrics and Health (GRECS), University of Girona.

#### 2.2.2. Homes

A web scraping of the Habitaclia website was carried out to determine the number of rental homes available in Catalonia. After reviewing the main rental platforms in Spain the Habitaclia portal was chosen because it is not only one of the platforms with the largest number of homes registered on it, but its web structure also allowed greater ease of data extraction for subsequent processing and geolocation.

##### Web Scraping

**Figure 5:**
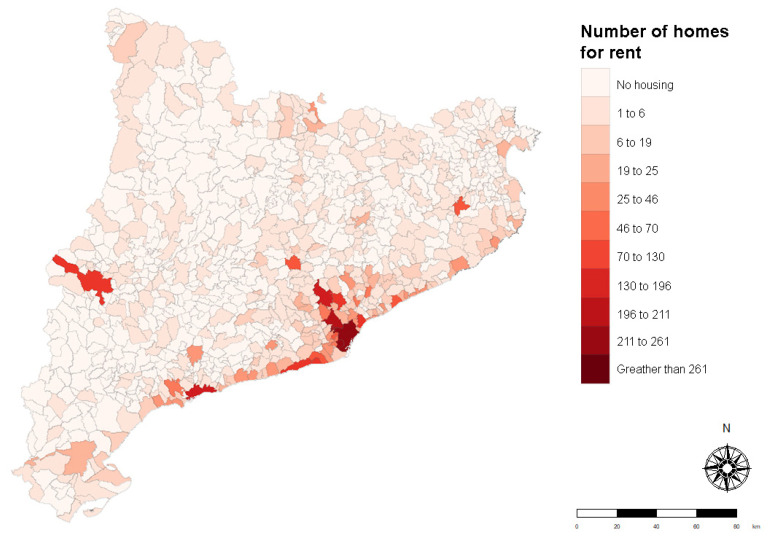
Map of number of homes for rent.

**Figure 6:**
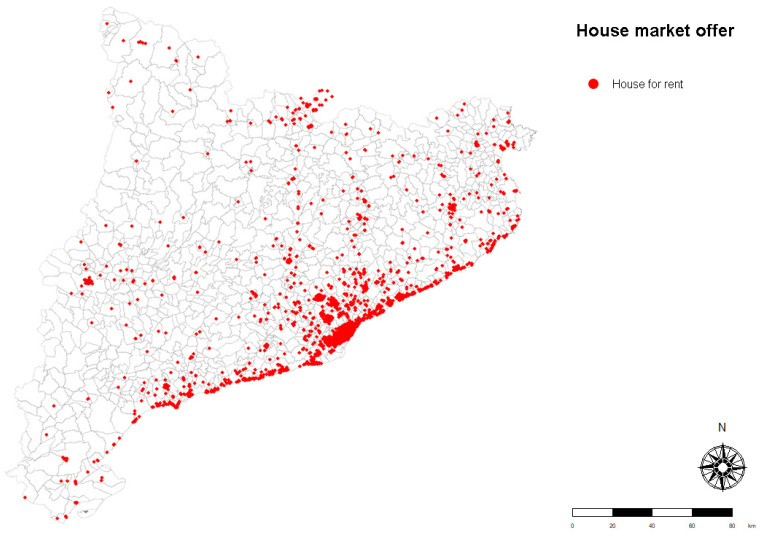
Map of real estate rental offer (each point is a house).

Web scraping (a technique using software to extract public information from websites in an automated way) allowed the following features of each home to be obtained: price, m^2^, number of toilets and rooms, province, municipality, district, and street (where the house is located). The total number of rental homes in Catalonia was 12,796, comprising 11,320 homes in the province of Barcelona (mean: 58.35 homes, standard deviation: 566.23 homes, median: 4 homes, first quartile -Q1-: 2 homes, and third quartile -Q3-: 15.75 homes); 582 in the province of Girona (mean: 4.89 homes, standard deviation: 10.48 homes, median: 2 homes, first quartile -Q1-: 1 home, and third quartile -Q3-: 4.5 homes); 234 in the province of Lleida (mean: 4.03 homes, standard deviation: 17.82 homes, median: 1 home, first quartile -Q1-: 1 home, and third quartile -Q3-: 1 home); and 660 in the province of Tarragona (mean: 10.82 homes, standard deviation: 28.37 homes, median: 3 homes, first quartile -Q1-: 1 home, and third quartile -Q3-: 3 homes). Figure 5 and Figure 6 shows the result for geolocalised homes in the area of study.

##### Geolocalisation, Atypical Values, Errors, and Debugging the Web Scraping

The data obtained from the web scraping were used to localise each home in its town and district of reference. Of the total number of homes obtained (*n* = 12,796), *n* = 11,289 were successfully geolocated. This debugging reported the following errors: platform errors (*n* = 3); homes with some missing information (*n* = 42); and homes where the geolocated municipality and province do not match (*n* = 142). All homes that were atypical due to their high rental price and could affect the objective of the study were also discarded (*n* = 1320).

#### 2.2.3. Variables

##### Dependent Variables

We considered, as a dependent variable, a variable indicating that, in September 2021, the home was not rented (value 1 and 0 if it was rented).

##### Explanatory Variables

− *Environmental and demographic data*

The cultural diversity [41] of the people living in a neighbourhood or area determines the socioeconomic level of its population. This socioeconomic level directly affects the area or neighbourhood, generating either favourable economic growth or reversing this growth [42].

The data were obtained taking the district in each town where the home was located as the reference. If they were subject to statistical secrecy, the value for the town itself was used. The variables used to determine the socioeconomic fabric of the territory were: average age, average size of homes, number of single-person households, and the Gini index [43].

− *Vegetation and the presence of greenery*

The relationship between the presence of parks, gardens, trees, and other green elements has been scientifically proven [44,45,46] to be linked to health [47]. The NDVI [48] and the surface area of green areas per inhabitant in square metres [49] were used to study the relationship between the homes and the surrounding greenery. The WHO recommendation regarding the latter is that every city should have at least 9 m^2^ of greenery per inhabitant [21].

In addition, the relationship between the presence of urban vegetation and the quality of life and health is known. There is also a relationship between urban vegetation and physical activity and mental health [50].

To observe the nearby NDVI of each house, a 500-metre buffer was constructed [51], where each house acts as a centroid of its own buffer. In this way, the vegetation close to each house can be analysed.

− *Air pollutants*

In recent years, there has been an ever expanding amount of scientific evidence concerning increased levels of pollution [52,53,54]. Consequently, pollution is highly relevant when considering the development of a healthy lifestyle.

The following variables regarding air pollution [55,56] were used: particulate matter equal to or less than 10 microns (PM_10_), nitrogen dioxide (NO_2_), and ozone (O_3_). In 2021, the WHO recommended new levels of contamination [57]. Families living in areas below these pollution levels will see their cardiovascular and respiratory health improve, in addition to reducing their burden of morbidity and mortality. We created two dummy variables for each pollutant and looked at whether homes were in locations where air pollution was above or below the old and current WHO limits.

− *Safety*

The safety of a town or area is an important element in determining its socio-economic status [58]. Low-income areas are directly related to different types of crime [59]. Although Europe in general does not have such a high or significant ratio as other countries [60], it is still an element to consider, especially for families who rent [18]. The number of crimes is also related to the movement of drugs, gangs, and other elements that can impact an area and condition the lives of the families living there [61].

The total number of crimes known to the Catalan police force (Mossos d’Esquadra) in the Basic Police Area (ABP) of each home was used [62]. The Mossos d’Esquadra are responsible for citizen safety, public order, investigation, and traffic control in Catalonia.

− *Ability to pay*

The rental price of homes determines the type of families who will be able to live in an area [63]. Each small area (either municipality or district) has similar prices for a home, which vary depending on the features, the socioeconomic level of the neighbourhood, the presence of nearby urban greenery, and how safe the area is. Migratory movements also affect the socioeconomic levels of the population [64].

The threshold for Catalonia (EUR 11,365.60) [65] was used to determine which flats could be rented by families at risk of poverty. The social structure of the territory was also obtained through the variables presented above. We have controlled the neighbouring areas of each house to observe their closest socioeconomic environments. We do so to observe the interaction of each dwelling with the areas close to them.

The variables linked to income to determine the ability to pay were net income per person per household, median per unit of consumption, and average salary [43]. The percentages of people at risk of poverty (60% of the median) and extreme poverty (40% of the median) were also obtained.

##### Control Variables

We controlled for different types of variables: features of the home, social characteristics, population size, and typologies of areas. More specifically, the area in square meters, and the number of rooms and toilets were used to consider the different features. In terms of population characteristics, we used the number of inhabitants, the average size and age of the home, and the population density in the district where each home was located.

The differences presented in the small areas (either municipality or district) forced the creation of multiple control variables to capture the heterogeneity of the territory: size of each district in square kilometres (source: extraction of the area through the municipal raster [66]); the variable density_urban, which measures the density of the urban areas of each town (source: data from the urban map of Catalonia, [49]); the variable area_metro, which captures whether or not the district in question belong to the metropolitan area of Barcelona (source: authors’ own); the variable capitals (source: authors’ own) to capture whether or not the district belongs to a provincial capital; and the variable density_ (source: authors’ own), which measures whether the district is in a high- (High) or low- (Low) density area, depending on the reference population. Apart from the variable density_urban, the variables were observed at the district level.

#### 2.2.4. Geospatial Groupings

The data were placed into four groups. The first set of data was georeferenced and applied to the characteristics derived from the dwellings as well as the NDVI and the dummy control variables. The second set was grouped by district or town depending on the availability of the information. The data in this second group correspond to the socioeconomic data and the dichotomous variables of rural/urban areas and belonging to the metropolitan area of Barcelona. The inhabitants by districts are distributed as follows: mean: 6167 inhabitants, standard deviation: 16,556.16 inhabitants, median: 1730 inhabitants, first quartile -Q1-: 433 inhabitants, and third quartile -Q3-: 6251 inhabitants. By municipality they are distributed as follows: mean: 16,378 inhabitants, standard deviation: 258,190.7 inhabitants, median: 961 inhabitants, first quartile -Q1-: 322 inhabitants, and third quartile -Q3-: 3903 inhabitants.

The third type of grouping included the Basic Health Areas (ABS), which are defined as the territorial units through which the primary health care services are organized [67]. These can have different dimensions depending on the accessibility of the population to be served. The variables in this third grouping consist of the air pollution data. The population distribution of the ABS is: mean: 20,636 inhabitants, standard deviation: 9192.051 inhabitants, median: 20,638 inhabitants, first quartile -Q1-: 14,018 inhabitants, and third quartile -Q3-: 26,558 inhabitants.

The last grouping includes the Basic Police Areas (ABP), which are defined as the basic territorial implementation units, themselves defined using territorial and police criteria [64]. The data in this last group are those related to the number of crimes. The distribution of people of in the ABP is: mean: 381,153 inhabitants, standard deviation: 577,201.9 inhabitants, median: 129,374 inhabitants, first quartile -Q1-: 65,844 inhabitants and third quartile -Q3-: 258,179 inhabitants.

### 2.3. Data Analysis

We specified generalised linear models (GLM) with variable response of a Bernoulli distribution, according to the possibility of renting a home, i.e., a variable indicating that, in September 2021, a specific home (of the 11.289 homes analysed) was not rented.

We included as the following explanatory variables in the GLM: the percentage of the population at risk of social exclusion in the district of each home; the vegetation index (also in the district); the number of m^2^ of green area per inhabitant in the town of each home; dichotomous variables indicating if the values of the air pollutants (PM_10_, NO_2_ and O_3_) predicted in the district exceed the limits defined by the WHO; and the number of known crimes in the district of each home. We also controlled for the control variables defined above.

For the effect of the metropolitan area on the possibility of a family at risk of social inclusion renting a home, the model was re-estimated, including the interaction of the metropolitan area.

Details are shown in the Supplementary Material.

The values of the coefficients and the Odds Ratios generated through the model were used to study which variables affect the event of households at risk of poverty renting a home.

We included the variables linearly (that is, the response of the dependent variable is the same to an increase in the variable, regardless of the level of the variable) and non-linearly (that is, the response of the dependent variable to an increase in the variable will depend on the level of the level of the variable) in the model, testing different reference categories to see how they interacted with the event of households at risk of poverty renting a home.

The models were compared using the Akaike Information Criterion (AIC), which reviews and penalises the flexibility of the model [68] and the control of multicollinearity.

### 2.4. Software

The processes of web scraping and geolocalisation were carried out using Spyder software (version 5.1.5) [69] based on the Python language. The libraries used were random, time [70], requests [71], IPython [72], bs4 [73], datetime [74], csv [75], pandas [76], os [77], re [78], math [79], msvcrt [80], tabulate [81], tkinter [82], tqdm [83], and googlemaps [84].

Obtaining and debugging the data and the statistical modelling were carried out with the software RStudio (version 4.1.3) [85], based on the R language. The libraries used were rgdal [86], rgeos [87], raster [88], tmap [89], BBmisc [90], haven [91], dplyr [92], and ggplot2 [93].

## 3. Results

### 3.1. Bivariate Analyses

The results are presented in Table 1 and Table 2 (bivariate analysis). Table 1 considers whether the home could be rented by a household at risk of social exclusion. Table 2 also considers the area where the home is located, either within the Metropolitan Area (AMB) or Outside the AMB (OAMB). The variables show quite asymmetric distributions, implying that only robust statistics (median and first and third quartile) should be used when interpreting the data.

The results of the bivariate analysis (Table 1) show that generally the properties that cannot be rented by families at risk of social exclusion tend to be found in areas with a high Gini index (Can rent: 32.9, Cannot rent: 36.1). It is also observed that the percentage of people at risk of social exclusion is lower (Can rent: 14.7, Cannot rent: 13.3). However, it should be noted that the percentage of families living in extreme conditions does not vary depending on whether the houses can or cannot be rented by households with adverse social conditions (Can rent: 7.7, Cannot rent: 7.7). Table 2 shows how, in both areas, the homes that cannot be rented by families at risk of poverty are in the areas with a higher Gini score (AMB—Can rent: 31.5; Cannot rent: 32.45. OAMB—Can rent: 34.1; Cannot rent: 36.1). It can also be seen how these homes are found to be in areas where there is a lower percentage of people at risk of poverty (AMB—Can rent: 16.3; Cannot rent: 15.4. OAMB—Can rent: 14.6; Cannot rent: 13.3). However, it can also be seen that the percentage of people below the poverty line is higher outside the metropolitan area than inside it. There is also a higher percentage of people in extreme poverty outside of the metropolitan areas. It can be observed that outside of the metropolitan area there are variations among the homes to which families with social difficulties and those that do not have access (AMB—Can rent: 7.9; Cannot rent: 7.6. OAMB—Can rent: 7.7; Cannot rent: 7.7).

The number of rental homes available for a family at risk of social exclusion that would allow them to save at least 30% of their income is just 9 out of the total of 11,097 homes (all of them outside the AMB). Income also shows how families with social limitations can live in areas where gross income per capita income is lower (Can rent: 41,990; Cannot rent: 54,708). If we look at the situation by area, the phenomenon is the same but the value for gross income per person and per household is higher within the AMB.

Green areas present no differences in terms of the homes that can be rented and those that cannot (Can rent: 7.847, Cannot rent: 7.847). There is, however, a difference regarding the NDVI near to the homes, with those that cannot be rented presenting a lower value (Can rent: 0.226, Cannot rent: 0.221). If we analyse the green areas, it is outside the metropolitan area that there are higher values of NDVI and green areas. Notably, when differentiating the areas it was observed that the homes that cannot be rented have higher NDVI values (AMB—Can rent: 0.285, Cannot rent: 0.312. OAMB—Can rent: 0.213, Cannot rent: 0.218).

In general, the homes that can be rented have lower levels of PM_10_ (Can rent: 23.567; Cannot rent: 23.893) and NO_2_ (Can rent: 26.358; Cannot rent: 27.922), and higher levels of O_3_ (Can rent: 51.111; Cannot rent: 49.978). However, when we looked at the different areas, it was detected that the levels of PM_10_ (AMB—Can rent: 23.081; Cannot rent: 22.914. OAMB—Can rent: 24.091; Cannot rent: 23.893) are reversed and are higher in the houses that can be rented by low-income families. Another way to analyse pollutant levels is by using the limits proposed by the WHO. If we take the former level recommended by this organisation, we can see that most properties are above the recommended PM_10_ levels. However, with the new WHO-recommended levels, the number of households with an optimal level of pollution is reduced, especially regarding NO_2_.

If we analyse the number of crimes, the analysis shows that this number is lower for properties that can be rented by families with financial difficulties (Can rent: 9780; Cannot rent: 14,303). When observed by area, it is outside the metropolitan area that families at risk of poverty live in homes where there are more crimes (OAMB—Can rent: 9121; Cannot rent: 8727). In contrast, in the metropolitan area, crime is higher in areas where homes can only be rented by wealthy families (AMB—Can rent: 14,303; Cannot rent: 15,074).

### 3.2. Results of the Estimation of the Generalised Linear Models (GLM)

Table 3 and Table 4 show the Odds Ratio of the GLM estimation models, with which we looked for the association between air pollutants, socioeconomic status, and the vegetation near a home and the possibility of not renting/renting by a family at risk of social exclusion. We controlled for socioeconomic and demographic variables and non-observed confounders for all these factors. Table 3 and Table 4 also show the 95% confidence intervals (95% ICr, as of now) and their *p*-value. The variables on income (per person and per household) were not significant in terms of the possibility of renting. Similarly, the new limits recommended by the WHO and the possibility to save were not significant.

There is a relationship between areas with a high percentage of people below the poverty line and the ease of renting a home for families at risk of vulnerability. The higher the percentage of population below the threshold, the lower the possibility of not being able to rent a home compared to areas with a lower percentage of population below the threshold. In the second quartile, the probability of a home not being rented decreases by 21.13% and by 48.61% and 57.79% in the third and fourth quartiles. With crimes, the phenomenon is reversed, the greater the number of crimes, it’s harder not to rent compared to homes in areas where there are fewer crimes. In the third quartile, the probability of not renting increases by 43.42% and in the quartile with the highest number of crimes, the probability increases by 320.64%.

Relationships were also observed between the possibility of not renting a home and pollutants. For the PM_10_ pollutant, the second and last quartiles are less likely not to be rented than the homes in the first pollution quartile. The odds decrease by 31.59% and 36.21%, respectively. For the NO_2_ pollutant, the probability of not being rented decreases to a lesser extent compared to the least polluted quartile. In the second quartile the probability decreases by 56.01%, and in the third and fourth quartiles the probability decreases by 19.09% and 18.66%, respectively. For O_3_, the higher the level of contamination, the greater the probability of not being able to be rented compared to less contaminated homes. In the third quartile the probability of not being rented increases by 58.26% and in the fourth quartile the probability is 62.28%.

In addition, if the homes are in areas above the old limits indicated by the WHO, the probability of not renting decreases by 46.85% if they exceed PM_10_ levels and by 28.77% if they exceed NO_2_ levels.

Regarding green spaces, for each increase of one m^2^ of green area per inhabitant, the probability of not renting the home will increase by 190%. If we look at the NDVI close to the home, the higher the vegetation index, the more difficult it is not to rent compared to homes in areas where there is a lower index. In the second quartile the probability increases by 19.46%, in the third quartile by 39.42%, and in the quartile with the highest number of crimes the percentage is a little lower at 31.55%.

If we carry out the study considering the areas where the homes are located, we can detect divergences between them. In this case, the study was performed with linear variables to facilitate interpretation and comparison.

The probability of not renting a home is reduced by 8.36% for every 1% increase in the population at risk of extreme poverty. The effect is different inside and outside the metropolitan area. Within the metropolitan area, the probability reduction is 19.05%. On the other hand, outside the metropolitan area the probability increases by 5.70%. Crimes have a similar effect in the two zones. For each crime that occurs, the probability increases by 0.005%. Within the metropolitan area it increases by 0.014%, while outside the metropolitan area the probability increases by 0.004.

If we look at pollutants, the effects are different for each 1 µg/m3 increase. For each increase of 1 μg/m^3^ in the PM_10_ pollutant, the probability of not renting decreases by 2.93%. We observed a different pattern between areas. In the metropolitan area, the probability decreases by 7.03%. Outside the area the probability increases by 8.08%. The NO_2_ pollutant had the opposite effect between areas to PM_10_. For each increase of 1 μg/m^3^, the probability of not renting increases by 0.38%. Within the metropolitan area the probability increases by 2.30%, outside the probability it decreases by 2.36%. For the pollutant O_3_, for each increase of 1 μg/m^3^ the probability of not renting decreased by 1.32%. Inside and outside the metropolitan area, the probability of not renting increases by 1.96% and 0.45%, respectively.

As for green areas, for each increase of 1 m^2^ of green area per inhabitant, the probability of not renting will also increase by 0.68%. Within the metropolitan area, the probability will increase by a greater magnitude, 4.17%. On the other hand, outside the metropolitan area the probability will increase by 0.75%. The nearby NDVI showed a different pattern to the m^2^ of green area. For every 1% increase in the index, the probability of not renting decreases by 78.21%. Within the metropolitan area the probability increases by 125.15% and, on the other hand, outside the metropolitan area the probability decreases by 60.35%.

## 4. Discussion

The reduction of poverty and related inequalities is one of the great struggles repeated cyclically throughout history. In recent years, it has been shown that increased productivity in not reflected in society in terms of salary increases or a greater ability to cover basic material needs [94]. We can also find in the literature multiple studies on where and how poverty is distributed [95,96,97] among the different social classes [98].

As has been shown, the lower and low-income social classes have higher mortality rates than the wealthy classes [99,100,101]. Although the aim of this study was not to determine the relative risk of mortality of families below the poverty line, we find significant relationships between the areas with the highest levels of pollutants, PM_10_, NO_2_, and O_3_, and the tendency for these families to live there. As is well known, the most disadvantaged social classes tend to have higher exposure to environmental pollutants than the rest of the population [102], causing a higher probability of developing diseases [103,104,105,106].

There are various articles that study the relationship between exposure to environmental pollutants and social class [107,108,109,110]. Most of them are based on the individual or the socio-economic status (SES) of the areas, and the authors relate these to the levels of environmental pollution recorded. As in some other studies [111,112], our work was carried out with reference to housing, subsequently linking this to the area where the homes are located. In Oslo [111], it was found that socially deprived neighbourhoods have a greater exposure to air pollution. Wheeler and Ben-Shlomo [112], analysing a survey from England, found that socially deprived neighbourhoods have higher exposure to air pollution. However, our consideration is the possibility of the home being rented to low-income families, based on the real market supply. Notably too, our study includes different types of cities, while previous studies have generally considered metropolises and major cities. Yet, despite these differences, the results are similar, only differing when we analyse our results by area type. In this regard, the similarities with previous studies are accentuated in the more urbanised areas and differ in the more rural areas. These differences may be due to variations in the type of territory, the vegetation levels, and the socioeconomic and demographic status of the territories studied. We must also consider the possibility that the new generations in Catalonia have changed their housing consumption patterns and prefer to rent rather than buy. In fact, the most recent studies [14] show that young people want to buy but cannot.

This difference between zones is repeated among various key variables, but with a reversed trend. Urban greenery per capita, for example, is a green variable of high interest to most major cities worldwide [113,114]. These cities stand out for their high population density [115,116], accentuating problems of access to vegetation, while the direct relationship between vegetation and health is well known. Even in large cities, policies and regulations are in force to increase and protect the number of square kilometres of green areas per capita and their vegetation [117,118]. There are several studies linking inequality and green areas [119,120]. As with previous studies, we found that an increase in square metres of green space per capita acts as a barrier to access for low-income families. The same phenomenon occurs if we look at studies of NDVI close to the home. When we look at the phenomenon in rural areas, the patterns of urban green space per capita and NDVI are reversed. It is the areas with less urban green space that generate access difficulties for families at risk of poverty. This phenomenon can be explained by the fact that there is a large volume of greenery in the rural areas of Catalonia. The evidence shows that historically there has been residential segregation, with poverty concentrated in zones or neighbourhoods [121,122]. These areas end up becoming communities of vulnerable people, perpetuating the poverty trap [123,124]. In our study, we observed this very trend. The higher the percentage of the population at risk of poverty, the more likely they are to rent these homes. Results show [125] that approximately 18% of European households and 10% of Spanish households have difficulty meeting their monthly payments. In our study, we found that there is a minimum population of 7.7% at risk of social exclusion who may present these difficulties, even in affluent neighbourhoods. These differences can be explained by the fact that in the present study we only considered data from our territory. If we differentiate the data by areas, we can see that in rural areas there is a low segregation of the population with low resources in the most precarious areas, which is not the case in urban areas.

The international literature shows that poverty is one of the most stable predictors of crime [126,127]. Moreover, the degree of urbanisation and safety are also directly related to the crimes committed [128]. Areas with greater social deprivation are associated with a higher level of crime [129,130]. Our results show that the most depressed areas have the lowest crime rates. If we look at behaviour in rural areas, it does reproduce the logic pointed out in previous studies. However, in urban areas, housing to which low-income families do not have access is found in the neighbourhoods with the highest crime rates. There are two possible reasons for these differences. They could be explained by the type of data identified as crimes, since there may be limitations when it comes to capturing data through administrative record channels. It could also be possible that crimes are not recorded in the place where they occurred, but rather in the place where the people who commit them live. We find it plausible to think that people who commit crimes do so more in rich areas than in poor ones.

Our study, unlike most of those mentioned above, performs an analysis of housing, allowing data to be obtained inside and outside large cities to see the similarities and differences. We also focused on small areas (either municipality or district), although this made the study more difficult because it meant creating different control variables. To this effect, we obtained data of multiple types that have an impact on inequalities in large cities and the most depopulated areas.

## 5. Conclusions

Out study may have some limitations stemming from its design. Firstly, the ecological inference fallacy must be considered when working with an ecological study. This fallacy complicates inferences at the individual level, given that there can be confounding elements inherent in the design typology. However, we tried to control the bias in the models as far as possible by including social, environmental, demographic, geographical, crime, and confounding variables, all of which were addressed at a small territorial level. It would be interesting to be able to carry out the study through individuals rather than through the possibility of renting to be able to verify whether these data are correct or, conversely, to show new variations in the inequalities.

Secondly, the processing of data on different scales may be hiding inequalities and altering the study. This could be especially pertinent with reference to rural areas where it is more difficult to obtain the same level of data granularity as in urban areas. Furthermore, a family living in one area does not necessarily share the same average value as another family in the area. This fact leads to a possibly random error of measurement. This explains why the data have been measured with error, because otherwise we would obtain inconsistent estimators [131]. Lastly, we observe that there is a greater volume of data in the Barcelona area than in other areas. Notably, this province is home to more than 73% of the total population of the territory.

We believe these limitations are offset by the strengths of the study. Firstly, we conducted a study using small areas that allowed district data to be analysed. Although we are not the only authors to conduct a study of small areas, the model responds well to both small and rural areas. Secondly, the models obtained used multiple observed and unobserved confounders. Thirdly, we combined an interesting set of variables that are closely linked to inequalities. All of them stem from poverty and although studied in a combined way, our model can respond to multiple intersectionalities. Fourthly, the data obtained study inequality through the market supply curve, allowing the reality of the housing market at any given time to be captured and its limitations to be observed, which would be more difficult if worked from the demand curve or the balance point.

Lastly, we obtained a model that encompasses different key aspects caused by poverty: environmental pollution, the poverty cycle and trap, crime, and vegetation in relation to small areas and of different types.

Although the present study only considers outdoor pollutants, it would be interesting to obtain a more accurate reading of the exposure of each home to indoor pollution. It would also be interesting to be able to cross reference the different variables in the model at the individual level and see how they develop in the different districts.It would be advisable to carry out a study at the Catalonia level to determine if there are areas that have already become or are in the process of becoming places where people with high or low purchasing power are concentrated in order to deconstruct these areas of poverty or wealth.

We think that this article can shed light on the housing problem in Catalonia and that our results can be used to adjust the different policies implemented in the fight against poverty. In the first place, inequality is not generated solely by a lack of economic resources. Policies on environmental contamination should be accelerated to reduce future morbidity problems in the population, which will culminate in affecting future public health policies. In addition, a way must be found to encourage and help low-income families to save so as not to fall into the poverty trap while ensuring a healthy environment to reduce the latent inequality present throughout the territory. These social, economic, and environmental policies must be resolved today so as to have a smaller, less significant impact on future policies and on the Catalan economy.

Finally, the model is easily reproducible at other scales, which makes it possible to reduce the ecological fallacy and also to be used in other countries.

## Figures and Tables

**Table 1:** Bivariate analysis according to the possibility of renting.

Housing to Rent		
Variables	Can Rent (n = 5174)	Cannot Rent (n = 5923)
Percentage of people with an equivalized disposable income below the risk of extreme poverty threshold (%)		
Mean (sd)	8.967 (3.979)	8.465 (3.941)
Median (Q1–Q3)	7.7 (6.5–10.6)	7.7 (5.6–8.2)
Min–Max	2–34.3	2–24.6
Percentage of people with an equivalized disposable income below the risk of poverty threshold (%)		
Mean (sd)	17.246 (7.096)	15.32 (7.116)
Median (Q1–Q3)	14.7 (12.3–20.9)	13.3 (9.5–15.5)
Min–Max	4.8–57.1	4.8–42.2
Gini Index (%)		
Mean (sd)	33.251 (3.905)	35.752 (3.759)
Median (Q1–Q3)	32.9 (30.5–36.1)	36.1 (33.1–38.3)
Min–Max	22.6–43.9	23.5–43.9
PM_10_ (μm/m^3^)		
Mean (sd)	23.476 (2.945)	23.989 (2.815)
Median (Q1–Q3)	23.567 (21.664–25.444)	23.893 (22.126–26.201)
Min–Max	12.172–34.057	12.172–34.057
NO_2_ (μm/m^3^)		
Mean (sd)	27.416 (7.546)	27.969 (7.517)
Median (Q1–Q3)	26.358 (23.053–30.19)	27.922 (23.186–30.19)
Min–Max	4.994–52.524	4.994–52.48
O_3_ (μm/m^3^)		
Mean (sd)	52.385 (8.096)	51.604 (7.542)
Median (Q1–Q3)	51.111 (46.078–57.988)	49.978 (45.469–57.929)
Min–Max	22.592–80.507	22.592–75.387
Vegetation vigour (NDVI) within 500 metres of the house (index between −1 and 1)		
Mean (sd)	0.252 (0.098)	0.235 (0.079)
Median (Q1–Q3)	0.226 (0.189–0.304)	0.221 (0.192–0.261)
Min–Max	−0.028–0.69	−0.019–0.69
Green areas per inhabitant (m^2^)		
Mean (sd)	14.738 (17.768)	11.913 (14.809)
Median (Q1–Q3)	7.847 (7.847–15.752)	7.847 (7.847–7.847)
Min–Max	1.955–346.709	0–273.822
Number of known crimes		
Mean (sd)	11,952,441 (6986.762)	15,356.411 (8702.922)
Median (Q1–Q3)	9780 (6498–15,510)	14,303 (8056–22,791)
Min–Max	341–27,890	467–27,890
Gross household income (EUR)		
Mean (sd)	46,423.656 (16,329.446)	57,562.09 (22,441.094)
Median (Q1–Q3)	41,990 (36,195.75–50,566)	54,708 (41,990–60,502)
Min–Max	20,489–105,681	26,291–132,268
Individual gross income (EUR)		
Mean (sd)	18,491.176 (6438.809)	23,198.498 (8409.087)
Median (Q1–Q3)	17,004 (13,966–20,606)	22,484 (17,004–24,532)
Min–Max	7218–39,760	8853–44,928
Can money be saved? (n—%-)		
Can save money	9—0.15%-	0—0%-
Cannot save money	5165—99.85%-	5923—100%-
Is it above the former WHO PM_10_ limit? (n—%-)		
Under limit WHO	617—11.05%-	338—6.05%-
Over limit WHO	4557—88.95%-	5585—93.95%-
Is it above the new WHO PM_10_ limit? (n—%-)		
Under limit WHO	4—0.07%-	1—0.02%-
Over limit WHO	5170—99.93%-	5922—99.98%-
Is it above the former WHO NO_2_ limit? (n—%-)		
Under limit WHO	4845—93.64%-	5512—93.06%-
Over limit WHO	329—6.36%-	411—6.94%-
Is it above the new WHO NO_2_ limit? (n—%-)		
Under limit WHO	55—1.06%-	10—0.17%-
Over limit WHO	5119—98.94%-	5913—99.83%-

**Table 2:** Bivariate analysis according to the possibility of renting inside or outside the metropolitan area.

Variables	Housing to Rent			
	Outside the Metropolitan Area (n = 2912)		Metropolitan Area (n = 8185)	
	Can Rent (n = 2182)	Cannot Rent (n = 730)	Can Rent (n = 2992)	Cannot Rent (n = 5193)
Percentage of people with an equivalised disposable income below the risk of extreme poverty threshold (%)				
Mean (sd)	8.733 (3.654)	8.206 (3.552)	9.138 (4.193)	8.502 (3.991)
Median (Q1–Q3)	7.9 (5.825–11)	7.6 (5.5–10.3)	7.7 (6.6–9.4)	7.7 (5.6–7.8)
Min–Max	2–34.3	2–20.4	2.9–19.4	2.2–24.6
Percentage of people with an equivalised disposable income below the risk of poverty threshold (%)				
Mean (sd)	17.513 (6.491)	15.983 (6.129)	17.05 (7.501)	15.227 (7.24)
Median (Q1–Q3)	16.3 (12.3–21.6)	15.4 (11.3–19.9)	14.6 (12.3–17.7)	13.3 (9.5–14.7)
Min–Max	4.8–57.1	4.8–34.3	6.6–35.9	5.6–42.2
Gini Index (%)				
Mean (sd)	31.705 (3.39)	33.142 (4.459)	34.379 (3.87)	36.119 (3.497)
Median (Q1–Q3)	31.5 (29.4–33.7)	32.45 (30.4–35.5)	34.1 (31.7–38.3)	36.1 (33.1–38.3)
Min–Max	22.6–43.9	23.5–43.9	24.4–41.5	25.5–41.5
PM_10_ (μm/m^3^)				
Mean (sd)	23.007 (2.987)	23.207 (2.744)	23.818 (2.866)	24.099 (2.808)
Median (Q1–Q3)	23.081 (21.285–24.769)	22.914 (22.007–24.734)	24.091 (22.126–25.897)	23.893 (22.126–26.446)
Min–Max	12.172–34.057	12.172–34.057	15.16–30.262	15.16–30.262
NO_2_ (μm/m^3^)				
Mean (sd)	27.17 (8.05)	26.346 (8.663)	27.595 (7.152)	28.197 (7.314)
Median (Q1–Q3)	26.397 (23.004–30.142)	26.45 (20.37–30.378)	26.35 (23.363–30.19)	27.922 (23.469–30.19)
Min–Max	4.994–52.524	4.994–52.48	13.52–52.419	13.52–52.419
O_3_ (μm/m^3^)				
Mean (sd)	54.702 (8.598)	55.679 (9.466)	50.694 (7.259)	51.032 (7.045)
Median (Q1–Q3)	55.031 (49.298–61.035)	56.67 (49.949–61.215)	49.743 (45.469–55.981)	49.966 (44.8–56.394)
Min–Max	22.592–80.507	22.592–75.387	37.651–71.425	37.651–71.425
Vegetation vigour (NDVI) within 500 metres of the house (index between −1 and 1)				
Mean (sd)	0.294 (0.119)	0.308 (0.131)	0.222 (0.064)	0.225 (0.062)
Median (Q1–Q3)	0.285 (0.207–0.367)	0.312 (0.207–0.385)	0.213 (0.179–0.245)	0.218 (0.191–0.242)
Min–Max	(−0.028)–0.69	0–0.69	0.003–0.578	(−0.019)–0.609
Green areas per inhabitant (m^2^)				
Mean (sd)	22.124 (22.272)	29.638 (30.838)	9.352 (10.747)	9.422 (8.138)
Median (Q1–Q3)	16.223 (12.041–25.786)	20.774 (12.041–31.746)	7.847 (7.847–7.847)	7.847 (7.847–7.847)
Min–Max	1.955–346.709	0–273.822	3.953–192.599	3.953–192.599
Number of known crimes				
Mean (sd)	8857.759 (4311.136)	8543.019 (3690.514)	14,209.326 (7667.583)	16,314.196 (8776.855)
Median (Q1–Q3)	9121 (5000–12,940)	8727 (5321–12,163)	14,303 (8056–22,791)	15,074 (8056–27,890)
Min–Max	341–15,535	467–15,535	3522–27,890	3522–27,890
Gross household income (EUR)				
Mean (sd)	42,140.024 (8797.947)	47,905.192 (11,046.034)	49,547.614 (19,534.271)	58,919.597 (23,287.825)
Median (Q1–Q3)	40,907 (36,194–47,221)	47,075 (39,387–54,708)	42,731 (36,574–54,962)	54,962 (41,990–71,287)
Min–Max	20,489–81,141	27,325–81,141	28,500–105,681	26,291–132,268
Individual gross income (EUR)				
Mean (sd)	16,252.919 (3319.883)	18,292.252 (4042.72)	20,123.489 (7572.718)	23,888.187 (8631.341)
Median (Q1–Q3)	15,900 (13,966–17,644)	17,490 (15,233–21,415)	17,659 (13,812–23,771)	23,771 (17,004–24,950)
Min–Max	7218–28,575	10,731–28,575	8987–39,760	8853–44,928
Can money be saved? (n—%-)				
Can save money	9—0.41%-	0—0.0%-	0—0.0%-	0—0.0%-
Cannot save money	2173—99.59%-	730—100%-	2992—100%-	5193—100%-
Is it above the former WHO PM_10_ limit? (n—%-)				
Over limit WHO	1859—85.20%-	663—90.82%-	2698—90.17%-	4922—94.78%-
Under limit WHO	323—14.80%-	67—9.18%-	294—9.83%-	271—5.22%-
Is it above the new WHO PM_10_ limit? (n—%-)				
Over limit WHO	2178—99.82%-	729—99.86%-	2992—100%-	5193—100%-
Under limit WHO	4—0.18%-	1—0.12%-	0—0.0%-	0—0.0%-
Is it above the old WHO NO_2_ limit? (n—%-)				
Over limit WHO	179—8.20%-	50—6.85%-	150—5.01%-	361—6.95%-
Under limit WHO	2003—91.80%-	680—93.15%-	2842—94.99%-	4832—93.05%-
Is it above the new WHO NO_2_ limit? (n—%-)				
Over limit WHO	2127—97.48%-	720—98.63%-	2992—100%-	5193—100%-
Under limit WHO	55—2.52%-	10—1.37%-	0—0.0%-	0—0.0%-

**Table 3:** Association between air pollutants and socioeconomic variables with the possibility of not renting a home for a family at risk of social exclusion.

Variable	UNADJUSTED			ADJUSTED ^1^		
	OR (95% CI)	Pr (>|z|)		OR (95% CI)	Pr (>|z|)	
Percentage of people at risk of poverty threshold [Quartile 1]						
Risk of poverty threshold Q2	0.6464 (0.5496–0.7601)	1.33 × 10^−7^	(***)	0.7887 (0.6481–0.9593)	0.017621	(*)
Risk of poverty threshold Q3	0.3884 (0.3426–0.4401)	<2 × 10^−16^	(***)	0.5139 (0.4362–0.6051)	1.55 × 10^−15^	(***)
Risk of poverty threshold Q4	0.2855 (0.2492–0.3268)	<2 × 10^−16^	(***)	0.4221 (0.3502–0.5082)	<2 × 10^−16^	(***)
Average value NDVI range 500 metres [Quartile 1]						
NDVI Q2	0.7982 (0.7013–0.9082)	0.00063	(***)	1.1946 (1.0044–1.4209)	0.044492	(*)
NDVI Q3	0.8224 (0.7243–0.9336)	0.00253	(**)	1.3942 (1.1679–1.6649)	0.000237	(***)
NDVI Q4	0.6191 (0.5437–0.7048)	4.32 × 10^−13^	(***)	1.3155 (1.0616–1.6312)	0.012317	(*)
Average PM_10_ [Quartile 1]						
PM_10_ Q2	0.7859 (0.6899–0.8953)	0.00029	(***)	0.6841 (0.5368–0.8716)	0.002139	(**)
PM_10_ Q3	1.1249 (0.9769–1.2955)	0.10209		1.0607 (0.8110–1.3867)	0.666568	
PM_10_ Q4	1.1275 (0.9615–1.3222)	0.13971		0.6379 (0.4802–0.8464)	0.001873	(**)
Green areas per inhabitant (m^2^)	0.9976 (0.9947–1.0005)	0.11222		2.9040 (1.7695–4.7033)	1.81 × 10^−5^	(***)
Is the average PM_10_ over or under the former WHO limits? [over limit]						
pm10_expounder limit WHO	0.4358 (0.3656–0.5189)	<2 × 10^−16^	(***)	0.5315 (0.4282–0.6589)	8.97 × 10^−9^	(***)
Average NO_2_ [Quartile 1]						
NO_2_ Q2	0.3887 (0.3418–0.4418)	<2 × 10^−16^	(***)	0.4399 (0.3770–0.5128)	<2 × 10^−16^	(***)
NO_2_ Q3	0.5584 (0.4788–0.6510)	1.05 × 10^−13^	(***)	0.8091 (0.6689–0.9783)	0.028884	(*)
NO_2_ Q4	0.5103 (0.4338–0.6001)	4.53 × 10^−16^	(***)	0.8134 (0.6729–0.9831)	0.032684	(*)
Is the average NO_2_ over or under the former WHO limits? [over limit]						
no2_expounder limit WHO	1.0207 (0.8359–1.2461)	0.84066		0.7123 (0.5630–0.9008)	0.004653	(**)
Average O_3_ [Quartile 1]						
O_3_ Q2	0.9915 (0.8670–1.1339)	0.90113		1.0416 (0.8929–1.2152)	0.604212	
O_3_ Q3	0.9834 (0.8533–1.1335)	0.81705		1.5826 (1.3398–1.8704)	6.88 × 10^−8^	(***)
O_3_ Q4	0.8432 (0.7188–0.9892)	0.03627	(*)	1.6228 (1.3386–1.9691)	8.75 × 10^−7^	(***)
Number of crimes [Quartile 1]						
Number of crimes Q2	1.4464 (1.2765–1.6393)	7.32 × 10^−9^	(***)	1.1324 (0.9578–1.3391)	0.145864	
Number of crimes Q3	1.4613 (1.2880–1.6586)	4.08 × 10^−9^	(***)	1.4342 (1.1794–1.7444)	0.000304	(***)
Number of crimes Q4	4.3761 (3.5951–5.3335)	<2 × 10^−16^	(***)	4.2064 (2.9383–6.0237)	4.31 × 10^−15^	(***)

(***) = *p* ≤ 0.001; (**) = *p* ≤ 0.01; (*) = *p* ≤ 0.05. Source: authors’ own elaboration. ^1^ Additionally, adjusted for the control variables.

**Table 4:** Association between air pollutants and socioeconomic and health variables and the possibility of not renting a home for a family at risk of social exclusion by area.

Variable	Adjusted			Adjusted ^1^			Adjusted ^1^		
	Non Filtred			Metropolitan Area			Out Of Metropolitan Area		
	OR (95% CI)	Pr (>|z|)		OR (95% CI)	Pr (>|z|)		OR (95% CI)	Pr (>|z|)	
Percentage of people at risk of extreme poverty threshold	0.916365(0.904883–0.927941)	<2 × 10^−16^	(***)	0.809471(0.786280–0.833023)	<2 × 10^−16^	(***)	1.056953(1.007717–1.106804)	0.02044	(*)
Average value NDVI range 500 metres	0.217895(0.120654–0.392645)	4.13 × 10^−7^	(***)	2.251505(0.870287–5.848026)	0.094835	(·)	0.396411(0.142164–1.099841)	0.07613	(·)
Average PM_10_	0.970661(0.950686–0.991048)	0.004992	(**)	0.929724(0.891240–0.969703)	0.000708	(***)	1.080775(1.031068–1.133166)	0.00125	(**)
Square metres of green area per inhabitant	1.006783(1.003523–1.010092)	4.64 × 10^−5^	(***)	1.041741(1.024869–1.059444)	1.30 × 10^−6^	(***)	1.007544(1.002478–1.012570)	0.00309	(**)
Average NO_2_	1.003829(0.995226–1.012518)	0.384383		1.023022(1.011949–1.034271)	4.31 × 10^−5^	(***)	0.976399(0.957402–0.995677)	0.01688	(*)
Average O_3_	1.013291(1.005693–1.020963)	0.000593	(***)	1.019605(1.009848–1.029496)	7.81 × 10^−5^	(***)	1.004532(0.989968–1.019289)	0.54348	
Number of crimes	1.000049(1.000042–1.000056)	<2 × 10^−16^	(***)	1.000142(1.000126–1.000159)	<2 × 10^−16^	(***)	1.000037(1.000005–1.000069)	0.02233	(*)

(***) = *p* ≤ 0.001; (**) = *p* ≤ 0.01; (*) = *p* ≤ 0.05; (·) = *p* ≤ 0.1. Source: authors’ own elaboration. ^1^ Additionally, adjusted for the control variables.

## Data Availability

All the data, including the code to produce the figures, can be requested from the first author (xperafita@dipsalut.cat).

## Supplementary Materials

The following supporting information can be downloaded at: https://www.mdpi.com/article/10.3390/ijerph20085578/s1, Suppelementary File S1. Methods. Supplementary Table S1. Variables.
